# Molecular characterization of cystinosis patients: predominance of the CTNS c.829dup mutation in Center of Tunisia

**DOI:** 10.1186/s12863-026-01411-z

**Published:** 2026-02-26

**Authors:** Chayma Sahli, Sameh Mabrouk, Nesrine Jemmeli, Salsabil Nouir, Roua Ltaifa, Taieb Messaoud, Hassen Ben Abdennebi, Salima Ferchichi, Mohamed Ghorbel, Sandrine Laradi, Latifa Chkioua

**Affiliations:** 1 Biochemistry Laboratory (LR00SP03), Bechir Hamza Children’s Hospital, Tunis, Tunisia; 2 Department Pediatric, Sahloul Hospital, Sousse, Tunisia; 3 Pediatric Department, Tahar Sfar University Hospital, Mahdia, Tunisia; 4 Faculty of Medicine of Monastir, University of Monastir, Monastir, Tunisia; 5 Research Laboratory of Human Genome and Multifactorial Diseases (LR12ES07), Faculty of Pharmacy, University of Monastir, Street Avicenne, Monastir, 5000 Tunisia; 6 Laboratory of Biochemistry, Farhat Hached Hospital, Sousse, Tunisia; 7 Department Ophthalmology, Farhat Hached Hospital, Sousse, Tunisia; 8 The Eurofins Biomedical Laboratory -Interlab, Toulouse, 31000 France

**Keywords:** Cystinosis, *CTNS* gene, Molecular genetics, Tunisian population, Pathogenic variants, c.829dup mutation, SNPs, Phenotypic variability, Genetic heterogeneity

## Abstract

**Background:**

Cystinosis is a lysosomal storage disease caused by the accumulation of intralysosomal cystine in different tissues and organs including: brain, cornea, kidneys, liver and, pancreas. This pathology is due the mutations in the *CTNS* gene that encode the cystinosin protein. In this retrospective study, we aimed to explore the genetic and phenotypic diversity of Tunisian patients with cystinosis in order to identify recurrent variants and their correlation with clinico-pathology features.

**Methods:**

Our retrospective study was conducted between 2022 and 2025 in collaboration with pediatric departments of Sahloul hospital of Sousse, Taher Sfar hospital of Mahdia and Hedi Chaker Hospital of Sfax, Tunisia. The entire coding region of CTNS-cDNA was screened to identify mutations and polymorphisms in 10 families: 5 unrelated and 5 related families with cystinosis. Screening for the common 57-kb deletion was performed by standard multiplex PCR, followed by direct sequencing of coding exons and their flanking exon–intron boundaries in the *CTNS* gene.

**Results:**

Molecular analysis of the *CTNS* gene was performed in affected individuals and their relatives, leading to the identification of six pathogenic variants. Two of them were previously reported in the Tunisian population: c.829dup (p.T277fs; rs752919200), c.971-1G > C (rs2142982540), c.1001 C > A (p.T334N; rs3375433), c.925G > A (p.G309C; rs758813679), and c.251delA (p.N84fs; rs1057516296). A novel frameshift mutation, c.529delA (p.N177Tfs) was also detected. The c.829dup variant was present in six homozygous patients (5 related and one unrelated) and one heterozygous patient. In total, 39 SNPs were identified, including ten novel variants that have not been reported in ClinVar. The most frequent was rs161400 (c.779 C > T; p.T260N), which co-occurred with all variants except with c.829dup; was also observed in heterozygous carriers of c.829dup exhibiting a severe cystinosis phenotype.

**Conclusion:**

Our findings underscore the potential phenotypic modulatory role of the rs161400 SNP, particularly in individuals carrying rare CTNS variants compared to patients carrying the recurrent c.829dup mutation. This study emphasizes the significance of the broader genetic landscape, including variant–variant interactions, in shaping the phenotypic heterogeneity observed among patients. Moreover, this study highlights the distinct mutational profile of Tunisian patients with cystinosis disease.

## Introduction

Cystinosis is a monogenic autosomal recessive lysosomal storage disorder caused by mutations in *CTNS* gene which encodes the lysosomal membrane cystinosin (UniProtKB/Swiss-Prot: O60931) that transports the lysosomal-free cystine from lysosomal to cytoplasm. The deficiency of cystinosin leads to free cystin accumulation in cells of various organs, especially the kidney, liver, cornea, thyroid, bone marrow, and spleen [1].

The disease is clinically classified into three different forms according to the degree of renal disease severity: nephropathic cystinosis or renal Fanconi syndrome, intermediate cystinosis, and non-nephropathic or ocular cystinosis [2, 3]. The three forms of cystinosis are caused by the pathogenic consequence of the genetic variations in *CTNS* gene.

The *CTNS* gene, (OMIM 606,272; GenBank NM_004937.2) is located on the short arm of chromosome 17 (13p) and contains 12 exons that are distributed across ~ 23 kb of genomic DNA. Cystinosin codes for a 367 amino-acid peptide, predicted to contain seven transmembrane domains (TM) topology and a tyrosine-based GYDQL lysosomal motif. The C-terminal tail is predicted to be oriented towards the cytosol and the highly glycosylated N-terminal region towards the lysosomal lumen [4, 5]. In addition, a conformational signal is situated in the fifth inter-TM loop [4].

Approximately 150 mutations [6] have been identified in patients with cystinosis, in patients with cystinosis including small deletions or insertions, missense, nonsense, splice site mutations in the coding regions or adjacent intronic regions, as well as mutations in the promoter region. In addition, several pathogenic mutations have been reported with geographic specificity. The most frequent *CTNS* mutation, which affects approximately 76% of Northern European alleles, is a large 57-kb deletion involving the first nine exons and part of exon 10 [7]. However, this deletion was not detected in Turkish, Tunisian or Iranian patients respectively in the respective studies, leading to the conclusion that its occurrence is limited in the Eastern Mediterranean and Middle East [5, 8, 9]. The most common mutation detected in Egyptian patients is c.829dup; p.T277fs but was completely absent in other studies conducted in the Middle East [10]. Other mutations such as c.681G > A (p.E227E) are very common in the Middle East (Turkey, Iran) and rare in Europe/North America [11]; splicing mutations, c.681G > A, c.681 + 1G > A have also been identified [12].

This present study aims to explore the relationship between the recurrent and rare *CTNS* gene variants and the phenotypic variability observed in Tunisian patients with cystinosis.

### Patients and methods

#### Patients

**Fig. 1:**
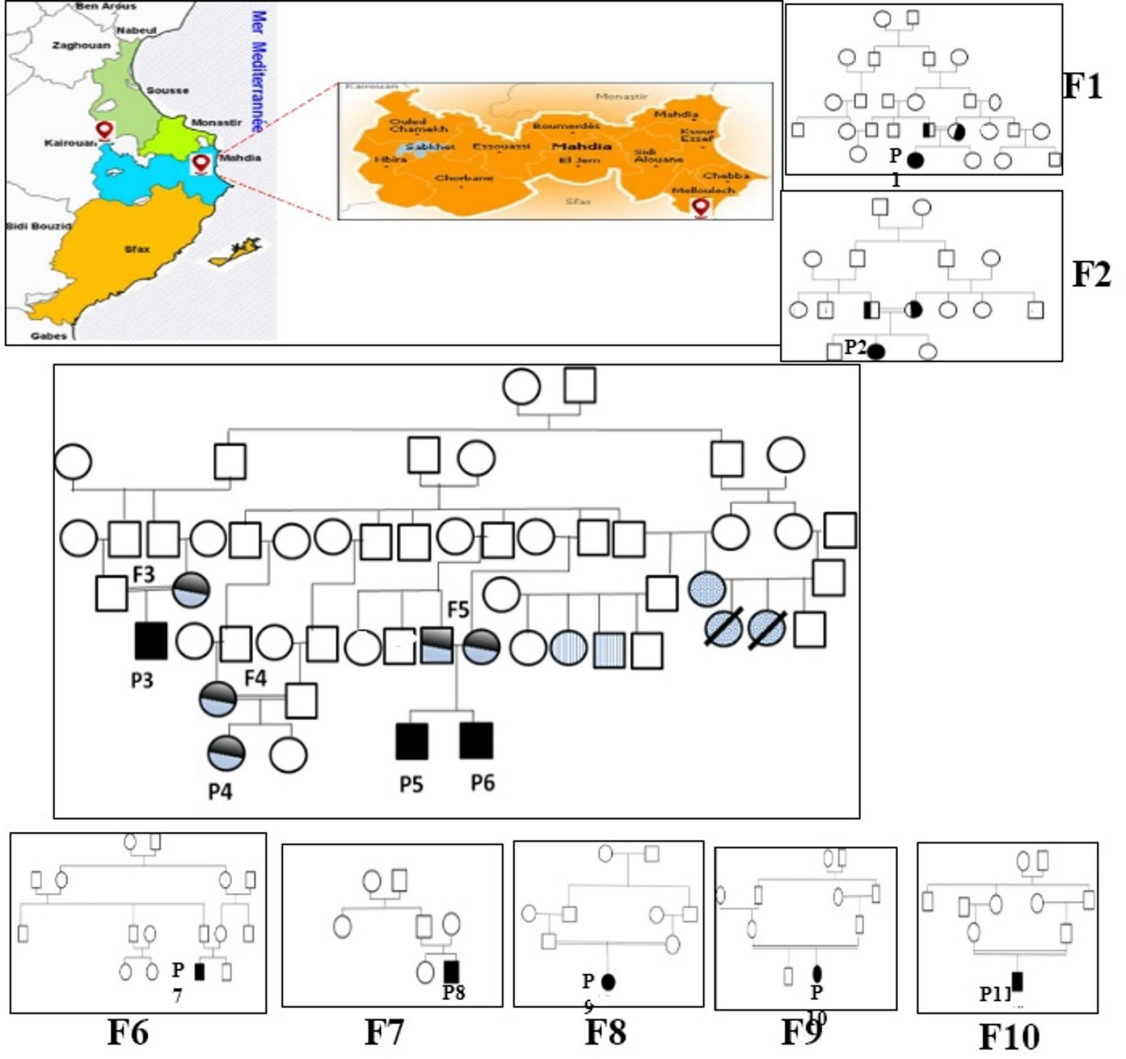
Pedigree of Tunisian patients with cystinosis disease. *Homozygous for p.T277fs and normal for T260N*. *Homozygous for p.Leu355P*. *Heterozygous for p.T277fs Heterozygous for T260N*. Geographic distribution and pedigrees of 10 Tunisian families affected by cystinosis.The map illustrates the regions of origin of the studied families, mainly located in central and coastal Tunisia. Pedigrees of families F1 to F10 are shown: Squares represent males and circles represent females.Black symbols indicate individuals homozygous for the p.T277fs variant and wild type for p.T260N. Dotted symbols correspond to individuals homozygous for the p.Leu355Pro variant presenting liver disease. Grey symbols indicate heterozygous carriers of the p.T277fs variant, while striped symbols represent individuals heterozygous for the p.T260N variant, affected individuals with associated heart disease. Slashed symbols correspond to deceased individuals. Consanguineous unions are indicated by double lines. The pedigrees highlight the autosomal recessive inheritance pattern and the segregation of disease-associated variants within and across families

This study is a continuation of previous research on Tunisian patients with cystinosis [5, 13], conducted between 2022 and 2025 included 10 families with 11 patients with cystinosis (P1–P11) who were diagnosed in different pediatric departments of Sahloul Hospital, Sousse, Taher Sfar hospital of Mahdia and Hédi Chaker Hospital of Sfax, Tunisia. A questionnaire had been elaborated in the Biochemistry Laboratory to collect epidemiological (sex, age, geographic origin and consanguinity) and clinical (family histories, clinical history, hospitalization, ophthalmic features, recurrent clinical symptoms and Serum electrolyte profile) data. All of these data are presented in Table 1. All investigated patients, except P8, were offspring of consanguineous marriages between first, second or third cousins from different areas of Tunisia. (Fig. 1). All affected children had healthy siblings, except for patient P5, whose brother P6 was also affected. Our cohort was divided into two groups according to consanguinity and geographical origin:

**Table 1:** Family history and clinical features of Tunisian patients with cystinosis

Families	F1	F2	F3	F4	F5		F6	F7	F8	F9	F10
Features	P1	P2	F3	P4	P5	P6	P7	P8	P9	P10	P11
Origin	Kairouan	Malloulich, Mahdia	Malloulich, Mahdia	Malloulich, Mahdia	Malloulich, Mahdia		Malloulich, Mahdia	Skhira, Sfax	Kasserine	Hajeb Laayoun, Kairouan	Zaghhouan
Consanguinity	Second degree	Second degree	Second degree	Second degree	Third degree		Second degree	Unrelated	Second degree	Second degree	Second degree
Age at diagnosis (year/month)	7 M	6 M	13 M	13 M	11 M	11 M	6 M	23 M	24 M	3Y and half	9 m
Age of onset (year/month)	12 M	6 M	12 M	6 M	10 M	11 M	5 M	Non precise	8 M	12 M	3 days
Age (years)	10Y	9Y	4Y,2 M	16Y	8Y	10Y	18Y	12Y	3Y	Died	5Y
Ophthalmic features	Fine retrocorneal precipitates, photophobia	Fine retrocorneal precipitates, photophobia	NL	NL	Fine retrocorneal precipitates, photophobia	Fine retrocorneal precipitates, photophobia	Fine retrocorneal precipitates, photophobia	Fine corneal sub epithelial deposits photophobia	No	No	Bilateral peripheral retinal lesions
Recurrent clinical symptoms	Growth retardation, dehydration rickets	Growth retardation, Recurrent vomiting associated with electrolyte disturbances	Growth retardation, polyuria and polydipsia	Growth retardation, psychomotor retardation	Growth retardation, dehydration rickets,	Growth retardation, dehydration rickets,	Dehydration, rickets, polyuria (10.2 ml/kg/h) and polydipsia	Growth retardation, rickets, motor retardation Polyuria (9 ml/kg/h) and polydipsia	Growth retardation, dehydration rickets, polyuria and polydipsia (10 cc/Kg/j)	Growth retardation, dehydration rickets, polyuria and polydipsia (15 cc/Kg/j) Recurrent Infectious disease	Growth retardation, rickets Recurrent lower respiratory tract infections, polyuria and polydipsia
Toni Debri Fanconi Tubulopathie	YES	YES	YES	YES	YES	YES	YES	YES	YES	YES	YES
Clinical course: End-stage renal disease on dialysis (ESRD)	ESRD at 5 years with start of hemodialysis	ESRD at 7 years with start of hemodialysis	No	No	ESRD at 7 years with start of hemodialysis	ESRD at 10 years with start of hemodialysis	ESRD at 7 years with start of hemodialysis	ESRD at the age of 7 years, started on peritoneal dialysis at the age of 10 years	CKD stage 3, conservative treatment	Deceased (severe dehydration / septic shock and catheter related thrombosis)	ESRD at age of 3 years, conservative treatment
Severe fatigue and psychomotor retardation	YES	YES	YES	YES	YES	YES	YES	No	YES	YES	YES
Serum electrolyte profile	Na : 130 mmol/l K : 2.8 mmol/l Bicabonate : 11.6 mmol/l Ca : 1.8 mmol/l Ph : 1.06 mmol/l Urines : NaU : 20 mmol/l Ku : 19 mmol/l CaU : 15 mg/kg/d PU : 42 mg/kg/d	None done	Ca : 2.3 mmol/l ph: 0.58 mmol/l Na: 135 mmol/l K: 3.19 mmol/l bicarbonate: 14.5 mmol/l Urine: PH: 6.5 G + + P+ caU/creatine ratio:0.25 PU: 13 mg/kg/d				Ca : 1.8 mmol/l Ph : 1.06 mmol/l Na : 130 mmol/l K : 2.8 mmol/l Bicabonate : 11.6 mmol/l Urines : NaU : 20 mmol/l Ku : 19 mmol/l CaU : 15 mg/kg/d PU : 42 mg/kg/d	Ca : 2.3 mmol/l, ph: 0.58 mmol/l Na: 135 mmol/l K: 3.19 mmol/l bicarbonate: 14.5 mmol/l Urine: PH: 6.5 G + + P+ caU/creatine ratio:0.25 PU: 13 mg/kg/d	Ca : 2,09 mmol/l, ph : 0,4 mmol/l, Mg : 0,5 mmol/L Na : 123mmol/l, K : 2,3mmol/l, Uric acid: 32 micromol/l Bicarbonate : 10 mmol/L Urine : PH = 5,5, G+++, P+ CaU/créatinine ratio : 0,33 PU : 20 mg/kg/d	Ca :1,51 mmol/L Ph :0,5 mmol/L Mg : 0,4 mmol/l Na :125 mmol/l K : 2,4 mmol/l Bicarbonate : 4,8mmol/L Urine : PH = 6, G+++, P++ CaU/creatinine ratio : 2,75 PU/creatinine ration : 150 mg/mmol	Ca : 2,1 mmol/L Ph : 1,7 mmol/L Na: 128 mmol/l K: 3 mmol/l bicarbonate: 14.5 mmol/l Urine: PH: 5,5 G + + P+ PU : 30 mg/kg/d CaU/ creatinine ratio : 2

- F2 to F6: share distant consanguineous ties and are originated from Malloulich locality, situated approximately at forty kilometers south of the town of Mahdia (Fig. 1). In this group, each family has one sibling except family F5 who have two siblings (P5 and P6) (Table 1).
- F1, F7 to F10: are unrelated families and are originated from different areas of Tunisia: Kairouan, Sfax, Zaghouan, Kasserine. Each family of this group has one sibling.
- It should be noted that patients P1, P2, P3, P4, P7, P9, P10, and P11 have second-degree consanguinity, while family F5, which contains two children (P5 and P6) with cystinosis, has third-degree consanguinity, whereas patient P8 from family F7 has no consanguinity.

#### Inclusion and exclusion criteria

We included 11Tunisian cystinosis patients with an average age of 9.5 years. The sex ratio was 1.75 in favor of boys. It should be noted that P9 died. The clinical profile was recorded including the severity of cystinosis, growth retardation, dehydration rickets, polyuria, polydipsia, myalgia with myogenic involvement, fine retro corneal precipitates, photophobia fine corneal subepithelial deposits photophobia, bilateral peripheral retinal lesion sand any family history of CTNS diseases. The diagnosis of disease was conducted by a pediatric clinician who confirmed the positive cystinosis diagnosis using standard protocols i.e. the assessment of frequency and severity of symptoms (patient/parents reported) and the use of objective the measurement of serum electrolytes such as sodium, potassium, bicarbonates, phosphate, renal function markers such as blood creatinine and urea levels, and features of proximal tubular dysfunction such as glycosuria without hyperglycemia, generalized aminoaciduria, low-molecular-weight proteinuria, and metabolic acidosis. Genetic testing is subsequently performed to confirm the diagnosis and identify the causative genetic variant, since the intra-leukocyte cystine quantification, considered as the gold standard for definitive diagnosis, is not available in Tunisian laboratories.

This study was approved by the Ethics Committee of the Sahloul Hospital (Sousse, Tunisia), and the families provided informed consent before collecting blood samples. All procedures were in accordance with the ethical standards of the responsible committee on human experimentation (institutional and national) and with the Helsinki Declaration of 1975, as revised in 2000 and approved by the Ethics Committees of the respective Tunisian hospitals.

### Methods

#### Ophthalmological examination

The Optical Coherence Tomography (OCT) of the cornea and retinal photography were used to search cystine crystals in corneas and conjunctiva in the most studied patients [5].

#### Molecular analysis

Peripheral blood samples were collected from all patients studied (P1–P11) who were followed in different pediatric departments, including Sahloul Hospital of Sousse, Taher Sfar Hospital of Mahdia, and Hedi Chaker Hospital of Sfax, Tunisia. Genomic DNA was extracted from peripheral blood leukocytes using the standard salting-out method, as previously described [14].

Molecular analysis of the *CTNS* gene was performed by amplification of the coding exons (exons 3–12) and their flanking intronic regions using previously primers [13], followed by direct Sanger sequencing to identify pathogenic variants and sequence polymorphisms. In parallel, multiplex PCR assays were carried out to screen for the recurrent 57-kb deletion of the CTNS gene, according to the protocol described by Chkioua et al. [5]. All sequencing procedures, data acquisition, and variant analyses were performed at the Laboratory of Biochemistry and Molecular Biology, Béchir Hamza Children’s Hospital, Tunis, using previously established and validated methodologies.

### Bioinformatics analysis

We used the PROTTER software V1.0 to predict the 2D structure and the functional impact of the different mutations (http://wlab.ethz.ch/protter/). Then, to evaluate the impact of the splice site mutation on mRNA slice mechanism, we used bioinformatic tools such as: Human Splicing Finder (HSF), and NNsplice (NNS).

## Results

### Nucleotide variations in the *CTNS* gene

**Fig. 2:**
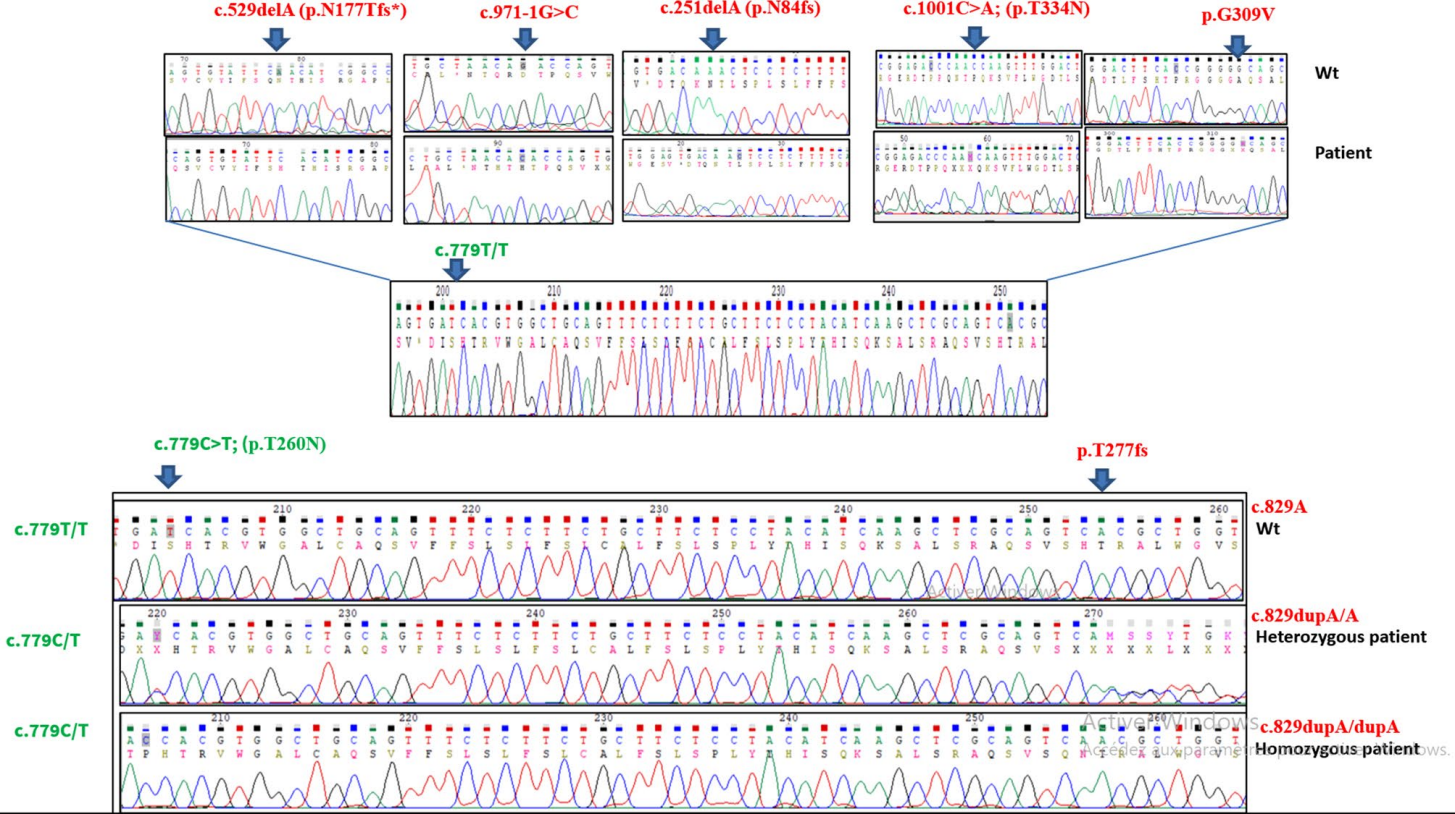
Sequencing profile of the identified mutations in analyzed patients showing the segregation of rs161400 and the pathogenic variant

Our analysis was limited to the coding regions of CTNS, based on the availability of primers in our laboratory, hence intragenic CNVs, deep intronic variants, or regulatory regions were not analyzed. We analyzed 11 Tunisian patients with cystinosis exhibiting mild and severe phenotypes. None of the patients carried the common European 57-kb deletion in the *CTNS* gene. DNA sequencing revealed a total of six different mutations (Fig. 2). Two of them were previously reported in the Tunisian population: c.829dup (p.T277fs; rs752919200), c.971-1G > C (rs2142982540), c.1001 C > A (p.T334N; rs3375433), c.925G > A (p.G309C; rs758813679*)*, and c.251delA (p.N84fs; rs1057516296). A novel frameshift mutation, c.529delA (p.N177Tfs) was also detected (Table 2).

**Table 2:** Genetic features in Tunisian patients with cystinosis

Families	Patients	Origin	Mutations	rs	SNP	Novel/ previously described
F1	P1	Kairouan	c.829dup. p.T277fs (H)	rs752919200	None	previously described
F2	P2	Malloulich, Mahdia	c.829dup. p.T277fs (H)	rs752919200	rs752919200(H), rs459613H, rs467277(H)	previously described
F3	P3	Malloulich, Mahdia	c.829dup. p.T277fs (H)	rs752919200	rs111526586 (H) ; rs112318471(H)	previously described
F4	P4	Malloulich, Mahdia	c.829dup p.T277fs (h)	rs752919200	rs161400(h)	previously described
F5	P5, P6	Malloulich, Mahdia	c.829dup. p.T277fs (H)	rs752919200	None done	
F6	P7	Malloulich, Mahdia	c.829dup. p.T277fs (H)	rs752919200	None done	
F7	P8	Skhira, Sfax	c.529delA; p.N177Tfs*(H)	**novel**	rs77453839(H) ; rs161400 (H) ; rs25713(H); rs459613 (H) ; rs467277(H) ; rs2507778658(H), 5,338,504(H), rs1323098109(h)	previously described
					c.62–216 C > G	**novel**
F8	P9	Kasserine	c.971-1G > C	rs2142982540	**rs2507795913(H)** **rs459613(H)**	previously described
F9	P10	Hajeb Laayoun, Kairouan	p.T334N(h) p.G309C (h)	ID3375433 rs758813679	rs2150925266(H); rs222753(h); rs1288637018(h) rs161400(H); rs1187529008(h); rs467277(h); rs457419(h); rs368132196 (H); rs2507698915(h); rs459613 (H); rs2076250370(h) ; rs2507793519(h)	previously described
					c.971–34 C > A (H) ; c.1085 + 64T > G (H) ; c.682–29 C > A(h) c.141-59delGT (H)	**novel**
F10	P11	Zaghhouan	p.N84fs (H)	rs1057516296	rs377470985(h); rs161400(H); rs2150925266; rs222753(h); rs459613(H); rs76153698(h); rs2150904273(h);; rs113967200(h);; rs2076241638(h);; rs2142981648(H); rs1233969942(H); rs76153698 (h); rs2150922708 (h); rs954472477**(h);**	previously described
					c.513 C > T ; L318L(H) ; c.971–57 C > A(H); c.628–29 C > A(h) ; C.461 + 11delT (H)	**novel**

The CTNS gene variants identified in our cystinosis patients are presented above. The table lists the pathogenic variants and associated single nucleotide polymorphisms (SNPs) detected in our patients. Variant nomenclature follows HGVS recommendations, based on the CTNS reference transcript NM_004937.3. Zygosity is indicated as homozygous (H) or heterozygous (h). Variant annotation and classification were performed using ClinVar and dbSNP. Variants designated as novel had not been reported in these databases or in the literature at the time of this study

The most frequent disease-causing mutation identified in the studied patients was p.T277fs, accounting for approximately 50% (11 out of 22) of the alleles. Five patients (P1-P3, P5 and her brother, P6 and P7) were homozygous for this mutation. At least one clinically affected patient (P4) carries only a single heterozygous CTNS c.829dup variant.

**Fig. 3:**
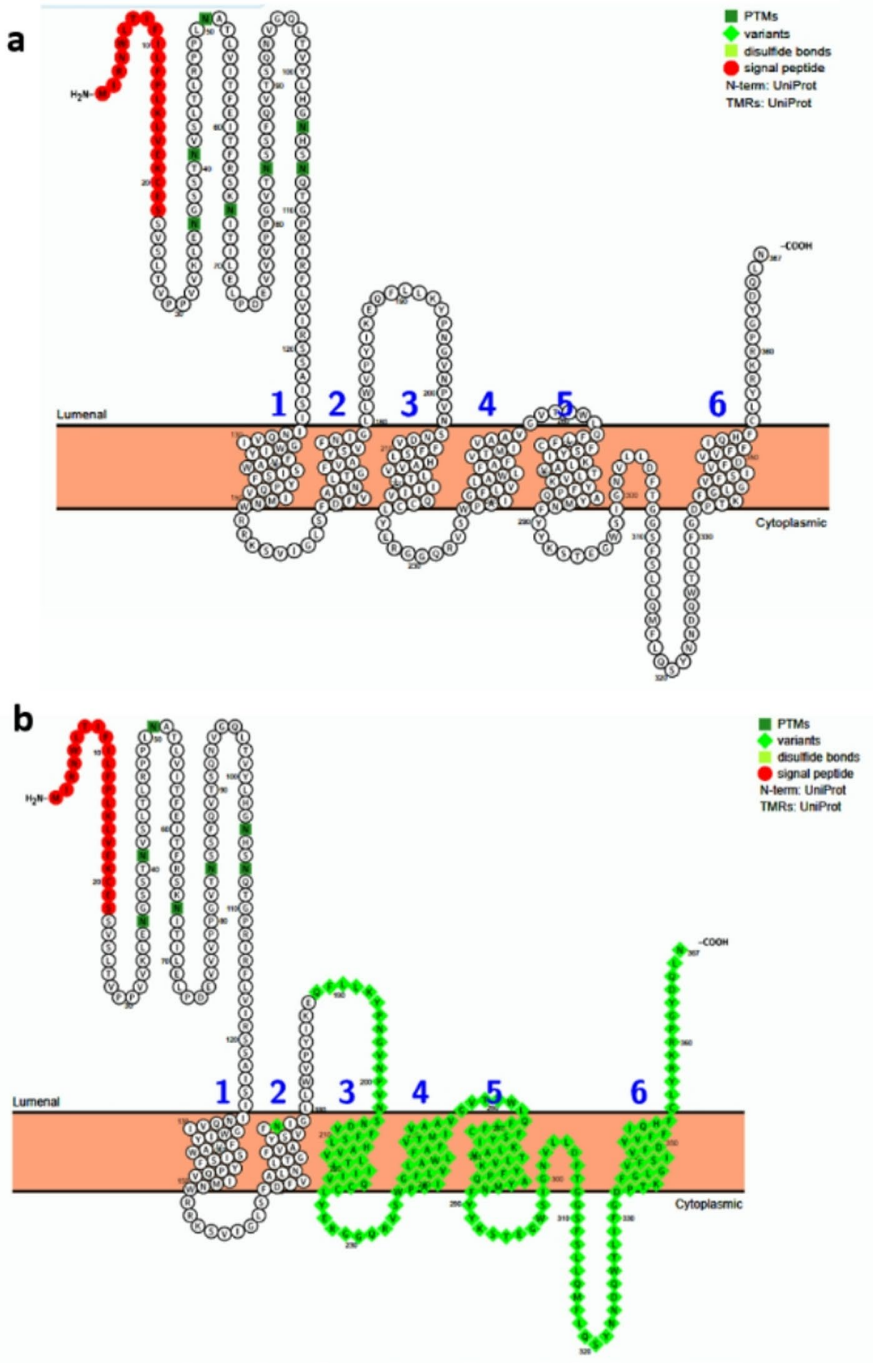
Predicted transmembrane structures of human cystinosin protein (UniProt O60931). **a** Human wild-type cystinosin protein. **b**: Effects of p.N177Tfs mutation on cystinosin protein predicted by the PROTTER program (http://wlab.ethz.ch/protter/)

The novel frameshift mutation p.N177Tfs, observed in homozygous state in patient P8, causes a premature termination codon at position 189, leading to the loss of the C-terminal region (residues 189–367), including several transmembrane domains essential for cystinosin function (Fig. 3).

The in-silico analysis of the splice-site mutation c.971-1G > C using HSF predicted the presence of a potential cryptic splice site near the natural acceptor splice site. According to NNsplice (NNS), the G > C substitution could abolish the canonical acceptor splice site (score = 0.98) and may activate a cryptic acceptor site at position c.971 + 10 with a predicted score of 0.85.

The screening of the *CTNS* gene revealed 39 single nucleotide polymorphisms (SNPs), including ten novel variants: c.513 C > T; L318L, c.461 + 11delG, c.971–57 C > A, c.628–29 C > A, **c.141 − 59_141-58delGT**, c.682–29 C > A, c.1085 + 64T > G, c.62–216 C > G, c.971–34 C > A.

We found that the most frequent polymorphisms, rs161400 was present in the homozygous state in patients and was associated with all identified mutation except p.T277fs frameshift mutation. The rs161400 was also detected in patient P4, who was heterozygous for the same mutation. Moreover, rs161400 co-segregated with all other genetic variants including: p.N177Tfs, c.971-1G > C, p.T334N /(p.G309C and p.N84fs, observed in the patients P8, P9, P10 and P11, respectively. The only exception were patients P1-P4 and P6, who were homozygous for the most common mutation, p.T277fs (Fig. 2) (Table 2).

## Discussion

This study represents the continuation of the largest Tunisian survey of cystinosis patients diagnosed during 2013–2025 [5, 13]. In Tunisia, the frequency of this disease is underestimated, considering the significant number of suspected cases through pedigree analysis of affected families. Our cohort includes 11 Tunisian patients who were investigated for the mutational molecular profile identification based on clinical and biological examination. The first, second or third degree of consanguinity was reported in all the studied families.

### Clinical data

The clinical presentation of Tunisian patients with cystinosis is highly variable (Table 1). Growth retardation was observed in all patients. End-stage renal disease (ESRD), the most prominent feature of cystinosis, was also observed in the most of patients (7/11). Dehydration and electrolyte imbalance due to renal tubular Fanconi syndrome were observed in all patients. However, cystine deposits were limited to the conjunctiva and retinal pigment epithelium in patients P1, P4, P5 (and her brother P6), and P7, according to several studies [5, 13]. Ophthalmological examination was not available in patients P3 and P4.

Patients P3 and P4 belong to two different families, F3 and F4, but both are derived from a large extended family. They exhibit distinct clinical manifestations, highlighting marked phenotypic heterogeneity within the sibship. Patient P3 presents with isolated growth retardation, whereas patient P4 shows psychomotor delay probably due to cysteine accumulation and profound asthenia, rickets, dehydration with symptoms fluctuating over time. All patients receive oral cysteamine treatment, except patient P8 who died at the age of 8 years.

Regarding hepatic and cardiac involvements, a definitive attribution to cystinosis cannot be established, since no biopsy was performed and no specific tests are available and leukocyte cystine level were not measured. For liver involvement, viral, toxic as well as autoimmune causes were ruled out. However, for cardiac involvement the involvement was retained in the absence of cardiovascular risk factors or associated heart abnormalities, with normal blood pressure. Cardiomyopathy due to cystinosis requires histological confirmation, which was not feasible in our patients, a causal relationship remains plausible given the lack of alternative identifiable causes.

### Genetics data

In our laboratory, the strategy for genetic analysis is initially based on the identification of the most common 57-kb deletion, even though it is rare in the Tunisian population, followed by DNA sequencing, targeting coding exons and exon–intron boundaries using previously published primers [5, 13] to identify the causative mutation. The variation annotation was performed using the RefSeq transcript NM_004937.4 and the genomic reference of CTNS (NG_012489.2) was used.

The most frequent mutation, 57 kb deletion, was absent in all Tunisian cystinosis patients. DNA sequencing revealed a total of six distinct mutations in which two of them were previously reported in Tunisian populations: c.829dup (p.T277fs; rs752919200), c.971-1G > C (rs2142982540), c.1001 C > A (p.T334N; rs3375433), c.925G > A (p.G309C; rs758813679). We also found c.251delA (p.N84fs; rs1057516296*)* and also one novel frameshift mutation, c.529delA (p.N177Tfs).

### c. 829dup mutation

All patients P1-P6 from families F1-F6 respectively, carrying the most frequent c.829dup mutation were homozygous for this pathogenic variation, except patient P4, (from family F4), who was heterozygous for it. This c.829dup (p.T277fs) mutation accounted for approximately 50% (11 out of 22) of all tested alleles. The Tunisian patients (P2-P6) were originated from related families residing in the same small region, Malloulich, in the governorate of Mahdia, whereas the patient P1 was originated from Kairouan, a governorate located approximately 107 km away from Malloulich.

The patient P4 from family F4 was found to be heterozygous for the most frequent mutation c.829dup but developed a severe clinical phenotype of infantile form of cystinosis. Sequencing of the entire cDNA-*CTNS* gene did not revealed additional pathogenic variation associated with the c. 829dup allele, nor was the common 57-Kb European deletion detected [7].

Following our clinical investigation, the patient (P4) has a similar clinical description with her mother including: renal involvement, myogenic impairment, manifested by myopathy, and was also affected by sensorineural hearing loss. On the cognitive level, intellectual disability was observed, as she demonstrated delayed comprehension and slow responsiveness. A similar spectrum of clinical features was observed within her family, including her sister, mother, and cousin suggesting the possibility of maternal inherence and the presence of secondary variants that may influence clinical progression. Given that both individuals (the mother and his daughter) exhibit a comparable clinical presentation, further studies will be necessary to confirm this hypothesis and to explore the possibility of a hypothetic additional maternally transmitted condition. In addition, the mother of patient P4 does not present clinical features of cystinosis and is heterozygous for the c.829dup mutation, whereas her daughter is affected despite also being heterozygous for the same variant. This observation suggests the possible presence of an additional pathogenic variant in an intronic or promoter region that was not analyzed in our analysis.

Moreover, the phenotypic analysis of this big family (including F3, F4 and F5) reveals a marked interindividual variability of phenotypic. In addition to the presence of an identical c.829dup mutation in all affected members, another genetic variation p.L355P, of FLII gene (OMIM; 600362) has also been detected in this extended family. The missense mutation p.L355P, which not yet been reported in ClinVar, is predicted to be probably damage (score = 1) with a PolyPhen.2 software (http://genetics.bwh.harvard.edu/ggi/pph2).

Therefore, the family F3, Family F4 and family F5, were subgroup of a large family who present a significant allelic heterogeneity. In particular, the presence of the c.829dup (p.T277fs) mutation in all patients with cystinosis.

Notably, several studies revealed that pathogenic mutations in FLII gene have previously been associated with dilated cardiomyopathy [15]. The coexistence of two different nonfunctional genes (CTNS and FLII) which could explain the clinical variability observed in this family. Although the pathogenicity of this variant must be confirmed by functional studies, its recurrence in a particular clinical context suggests that it could act as a modifying factor influencing both the severity and nature of the extra-renal manifestations of cystinosis.

Our study highlights the potential frequency of this c.829dup mutation in our population. This mutation may represent a founder effect, but further haplotype and population studies are needed. This finding is consistent with observations reported in other regions of North Africa and of the Middle East [10], where certain recurrent mutations in the *CTNS* gene appear to be linked to common ancestral lineages.

The c.829dup mutation has also been detected in Egyptian patients, considered as the most common CTNS variant and was found in the homozygous state in unrelated Egyptian families (6/26 or 23.1% mutant alleles) [10]. Notably, this mutation has only been observed in a heterozygous European patient [16].

It has been demonstrated that Tunisia shared with other North African countries (such as Morocco, Algeria) and Middle East countries (such as Egypt) a genetic diseases, mainly in the context of founder effects [17]. Furthermore, regional genetic databases have documented the distribution of inherited disorders across Arab populations, including Egypt [17].

Besides, the historical connections between Tunisia and Egypt, including migration, invasions, and trade, may contribute to the presence of shared genetic variants between these two neighboring populations. This knowledge suggests that the c.829dup mutation could represent a founder mutation in the both countries. A haplotype analysis in patients and control groups from Tunisia and Egypt would be important for further investigation.

Of note the c. 829dup (p.T277fs) mutation is deleterious, introducing a premature stop codon downstream that may trigger nonsense-mediated mRNA decay. This finding explains the lack of cystine transport activity. Several studies have shown a strong correlation between this mutation and the infantile nephropathic form of cystinosis [5, 10, 18].

### Novel frameshift mutation p.N177Tfs

The patient P8 originated from the Center of Tunisia was born from a non-consanguineous parent. He presented with classical nephropathic cystinosis and was found to be homozygous for the novel frameshift mutation (p.N177Tfs). The residu177 is located in the central region of the protein, near one of the inter-helical loops which is the third cytosolic loop according to a previous study [5]. Premature translation termination at residu187 would therefore eliminate the most of the C-terminal domains, including several posterior transmembrane helices and lysosomal C-terminal GYDQL targeting motifs which are essential for correct addressing and transport function.

The frameshift mutation p.N177Tfs results severe phenotype observed in patient P8 could be explained by potential impact of the premature termination codon at position 189, leading to the loss of five transmembrane domains essential for cystinosin function. several studies focus on functional and structural mapping of cystinosin indicate that the residue Asp305 plays a crucial role for proton coupling, substitutions at this position severely impair H⁺/cystine antiport [19]. Additional residues such as Ser228 shapes the binding pocket and is critical for efficient transport activity [20]. The resulting impairment of lysosomal cystine export correlates with severe clinical manifestation observed in patient P8 who’s homozygous for this mutation. The frameshift mutation is associated with an infantile nephropathic form (severe phenotype) with early renal involvement.

### Splice site c.971-1G > C mutation

The patient 9 (P9) was homozygous for the splice site c.971-1G > C mutation. According to the in-silico predictions, the wild donor splice site and the mutant splice site have a higher CVs and a below 80 respectively.

The G > C mutation at the 5′-acceptor splice site of intron 12 may lead to aberrant splicing and the inclusion of 10 intronic nucleotides in the mature mRNA which is expected to result in an abnormal, misfolded polypeptide (http://rulai.cshl.edu/tools/ESE3). This should explain the severe clinical features observed in patient P8. In further studies, we may suggest to perform an in vitro analysis in order to confirm the pathogenicity of this mutation and therefore to validate the impact of the splice site mutation in the process of precursor mRNA maturation. ClinVar (SCV001554457; rs2142982540) reports it as likely pathogenic. The splicing variant c.971-1G > C has previously been frequently reported in the central region of Tunisia [21]. Its identification in a patient (P9) from Kasserine, located approximately 230 km from this region, supports the hypothesis of a possible founder mutation. This observation is consistent with several studies reporting that the splice site mutations are rarely identified in patients with various forms of cystinosis [22–24].

A prenatal diagnosis could be conducted in the sixth family, showing that the fetus DNA was normal for the for this splice site mutation. Moreover, the antenatal diagnosis permits better understanding and a reliable genetic counseling in Tunisia, which is known for its big families and high-rate consanguinity.

### p.T334N and G309C missense mutations

The patient (P10) originated from the Center of Tunisia (Kairouan), presenting the infantile form of disease, was born from consanguineous parents. She was a compound heterozygote for the two previously reported missense mutations: p.T334N and G309C. She developed a severe clinical feature and died at the age of four. A previous study using a 3D structural model of cystinosin, generated through crystallographic analysis, demonstrated that most missense mutations associated with the infantile form cystinosis are located in the transmembrane domains [5]. These mutations impair the protein’s stability and affect the ability to transport cystine across the lysosomal membrane, which likely explains the severe infantile nephropathic phenotype observed in this patient [25]. These missense mutations in the transmembrane helices affect not only the ability of transporter to maintain H⁺-coupled transport and cystine passage, but also its stability, folding, and possibly targeting on the amount of functional protein at the lysosomal membrane. This may justify the severity of the observed clinical phenotype of this patient.

### Single nucleotide polymorphism p.T260N

in a previously study, we demonstrated that the unclassified sequence variant p.T260N (refSNP ID: rs161400) was co-segregate with several identified mutations (p.G308R, p.Q88K, c.681 + 7delC, p.S139Y) in Tunisian patients with cystinosis [5]. In this report, the p.T260N variant was found to be associated with the several mutated alleles (p.N177Tfs, p.T334N, G309C, p.N84fs), with the exception of homozygous patients for the c.829dup mutation (P1, P3, P4, P5 and P6). This finding suggests that the p.T260N variant may occasionally be present in individuals carrying the c.829dup mutation in the heterozygous state (as in patient P4), but it is consistently absent in patients who are homozygous for this mutation.

Interestingly, the patient P4, heterozygous for the c. **829dup** mutation that causes a premature stop, was also found to be heterozygous for the p.T260N variant. This situation indicates that the potential incompatibility between the two variations (p.T277fs and p.T260N) does not necessarily translate into heterozygosity for the frameshift mutation. The wild-type allele on the other chromosome of patient P4 may compensate for the loss of function induced by the premature stop codon, thus allowing tolerance of this genetic combination. This observation also suggests that the homozygous p.T260N is functionally well tolerated in case of association of heterozygous variants.

The single nucleotide polymorphism p.T260N may co-segregate with an alternative allele and is potentially excluded from the haplotype carrying the c.829dup vitiation in homozygosity. One likely hypothesis is that the p.T260N variant resides on a different ancestral haplotype than the one associated with c.829dup; that linkage disequilibrium or selective pressures might prevent their co-occurrence in a homozygous condition. This raises the possibility that p.T260N could proceed as a phenotypic modifier only in the presence of specific allelic backgrounds, which deserve further investigation through haplotype analysis and functional studies. Finally, the hypothesis of a phenotypic modifying role of the SNP cannot be excluded, particularly in patients carrying mutations other than the frequent mutation. The p.T260N polymorphism could, through subtle regulatory mechanisms (splicing, gene expression, protein-protein interactions), modulate the clinical expression of the disease, as has been demonstrated for other monogenic diseases.

Of note, in the present cohort, rs161400 appears to be a frequent variant (7/22), as does another polymorphism, rs459613 (10/22). In our previous studies, we demonstrated that rs161400 is commonly observed in Tunisian patients and represents the most recurrent polymorphism in this population (4/8) [5]. Further studies in larger cohorts and in other North African populations would be valuable to better assess its distribution and potential clinical relevance.

### Oral cysteamine treatment and prenatal diagnosis

In patients with cystinosis, it has been demonstrated that the accumulation of cystine crystals in renal tubular cells leads to an abnormal accumulation of autophagosomes in cells, marked by the LC3 biomarker [26]. This pathway activates the autophagic process, contributing to the progressive degradation of tubular cells, leading to renal damage. These findings are consistent with the clinical data observed in the present study. Most patients (P1, P4, P5, P6, P7, P8P8) have already started early initiation of dialysis.

Of note, new therapeutic strategies for cystinosis have been developed, including the transplantation of genetically modified hematopoietic stem and progenitor cells, as well as lipid nanoparticles, based on mRNA (LNP/CTNS) therapy. In the present report, most of studied patients are on oral cysteamine treatment, but, consequently, the molecular diagnosis remains the only strategy to confirm the diagnosis, because of the absence of leukocyte cystine measurement availability in the on hospital-institutional structure in Tunisia. The additional benefits of this molecular strategy are to prevent the birth of homozygous mutated children and to also to determine the genetic profile of affected families through detailed family pedigree analyses.

## Conclusion

This study highlights the importance of the overall genetic context and interactions between variations on order to better understand the phenotypic variability observed in patients and the role of founder mutations and genetic diversity on the severity of the observed clinical features. These findings emphasize the importance of international collaboration to identify founder haplotypes in populations that share the same recurrent mutation. Eventually, future research should focus on expanding genotype–phenotype correlations, understanding modifier variants, and addressing healthcare to optimize long-term outcomes for individuals with cystinosis worldwide.

## Data Availability

The datasets analyzed during the current study are available in the ensemble database (https://www.ensembl.org/index.html)The datasets analysed during the current study are available in the ensemble database(https://www.ensembl.org/index.html)- c.829dup (p.Thr277fs; rs752919200): [https://www.ncbi.nlm.nih.gov/clinvar/variation/551290/](https:/www.ncbi.nlm.nih.gov/clinvar/variation/551290)Variation ID: 551290Accession: VCV000551290.15- c.971-1G> C (rs2142982540): [https://www.ncbi.nlm.nih.gov/clinvar/variation/1065114](https:/www.ncbi.nlm.nih.gov/clinvar/variation/1065114) /Variation ID: 1065114Accession: VCV001065114.2- p.Thr334Asn (rs3375433): [https://www.ncbi.nlm.nih.gov/clinvar/variation/3375343/](https:/www.ncbi.nlm.nih.gov/clinvar/variation/3375343)Variation ID: 3375343Accession: VCV003375343.1- c.925G> A (p.Gly309Cys; rs758813679: Submission: [https://submit.ncbi.nlm.nih.gov/subs/variation_clinvar/SUB15775330/](https:/submit.ncbi.nlm.nih.gov/subs/variation_clinvar/SUB15775330)Submission ID: SUB15775330Organization ID: 507525- c.251delA (p.Asn84fs *;* rs1057516296 *):* [*https://www.ncbi.nlm.nih.gov/clinvar/variation/370183*](https:/www.ncbi.nlm.nih.gov/clinvar/variation/370183) */*Variation ID: 370183Accession: VCV000370183.4- c.529delA(p.Thr177fs): Submission: [https://submit.ncbi.nlm.nih.gov/subs/variation_clinvar/SUB15775414/](https:/submit.ncbi.nlm.nih.gov/subs/variation_clinvar/SUB15775414)Submission ID: SUB15775414Organization ID: 507525- c.779 C> T (p.Thr260Ile) ; rs161400 : [https://www.ncbi.nlm.nih.gov/clinvar/variation/257157/](https:/www.ncbi.nlm.nih.gov/clinvar/variation/257157)Variation ID: 257157Accession: VCV000257157.35.
